# Hypocalcemic cardiomyopathy with heart failure: A rare Case report

**DOI:** 10.1002/ccr3.9463

**Published:** 2024-09-22

**Authors:** Manish Kharel, Anukul Subedi, Md Fahad Hossain

**Affiliations:** ^1^ Kathmandu medical college Sinamangal Nepal; ^2^ Ministry of Health and Family Welfare Kuliarchar Kishoreganj Bangladesh

**Keywords:** cardiomyopathy, case report, heart failure, hypocalcemia, hypoparathyroidism

## Abstract

Hypocalcemia is one of the rarest causes of reversible cardiomyopathy. Patients with refractory heart failure need to be explored for hypocalcemia and need prompt correction.

**Abstract:**

Hypocalcemia is a rare cause of reversible dilated cardiomyopathy. Correction of calcium is crucial to recover left ventricular function and structure. We presented the case of a 55‐year‐old female who was admitted to the hospital with refractory heart failure due to hypocalcemia induced by primary hypoparathyroidism and complicated by vitamin D deficiency. The patient's cardiac symptoms improved dramatically upon correction of hypocalcemia, and vitamin D. Therefore, the key clinical message of this case report is, that hypocalcemia, although rare, should be considered as one of the differential diagnoses when heart failure is refractory and early diagnosis and treatment is necessary as it is the cause of reversible cardiomyopathy and could reduce morbidity and mortality.

## 1 INTRODUCTION

Calcium ion is responsible for myocardial contractility through a process called excitation and contraction coupling. An increase in cytosolic calcium activates the cross‐bridge formation between myofilament protein that causes the myocardial contraction following the ejection of blood and relaxation occurs when cytosolic calcium pumps back to the sarcoplasmic reticulum unbinding the cross bridge.^1^

Hypo‐calcemia followed by dilated cardiomyopathy is a rare but potentially reversible condition. Despite the common occurrence of hypocalcemia and hypoparathyroidism, hypo‐calcemic cardiomyopathy is rare, with only 61 cases being reported in one literature review.^3^ The exact pathophysiology of hypo‐calcemic cardiomyopathy has not been yet clarified; the lack of calcium ion mechanism hypocalcemia described above is thought to be one.^2^ 87% percent of cases in hypocalcemia‐induced cardiomyopathy is heart failure with reduced ejection fraction, a rare case of heart failure and the manifestation could vary from asymptomatic to life‐threatening hypotension, arrhythmia, and dilated cardiomyopathy as in our case.^3^

Cardiomyopathy is further complicated by hypomagnesemia and low vitamin D levels which also need to be monitored and treated.^4^, ^5^ The prognosis of hypo‐calcemic‐induced cardiomyopathy is good with restoration of normal cardiac function on normalizing calcium.^6^ We present the case of hypo‐calcemic cardiomyopathy caused by primary hypoparathyroidism and complicated by vitamin D deficiency.

### 1.1 Case history and physical examinations

A 55‐year‐old female with no past medical and family history came to the hospital with the chief complaint of shortness of breath for 1 day. She complained she used to have exertional dyspnea, sometimes dyspnea at rest, for the last 6 months. At admission, the patient was conscious but with severe respiratory distress, normal body temperature, peripheral saturation of 70 percent, B.P 100/60 mm hg, respiratory rate of 25 breaths per min, and pulse rate of 110 beats per min. On Physical examination, bilateral basal crackle, and hepatomegaly were present.

### 1.2 Investigations, diagnosis and treatment

Important initial investigation reports are, HB: 10 g/dL, Sodium:132 mmol/L, Potassium: 4 mmol/L, Urea: 106 mg/dL, Creatinine: 3.4 mg/dL, and Pro BNP: 1500 pg./mL.

**FIGURE 1:**
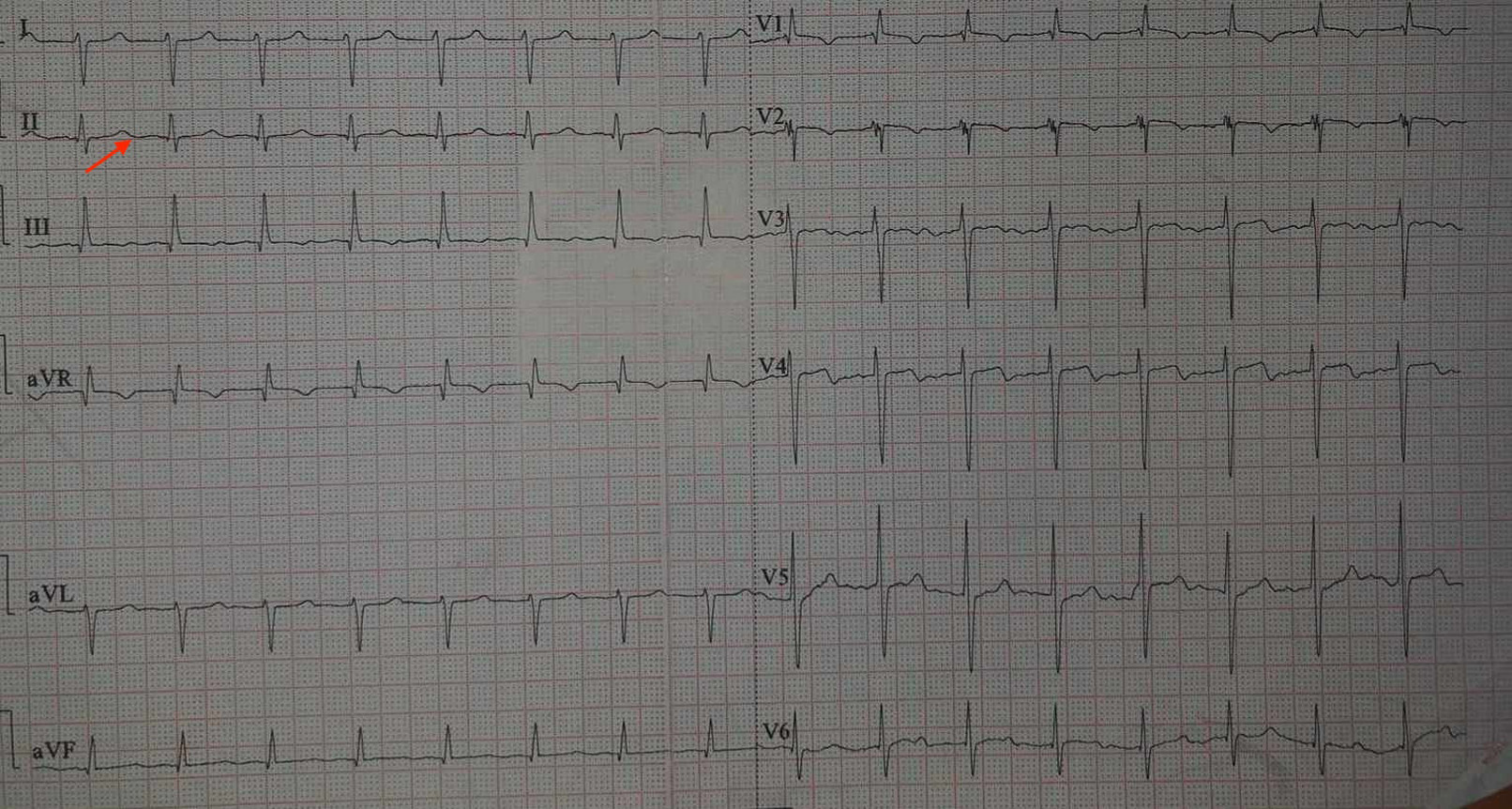
ECG shows sinus tachycardia of rate 115 bpm with long QT with QTc: 498 ms (marked by arrow).

**FIGURE 2:**
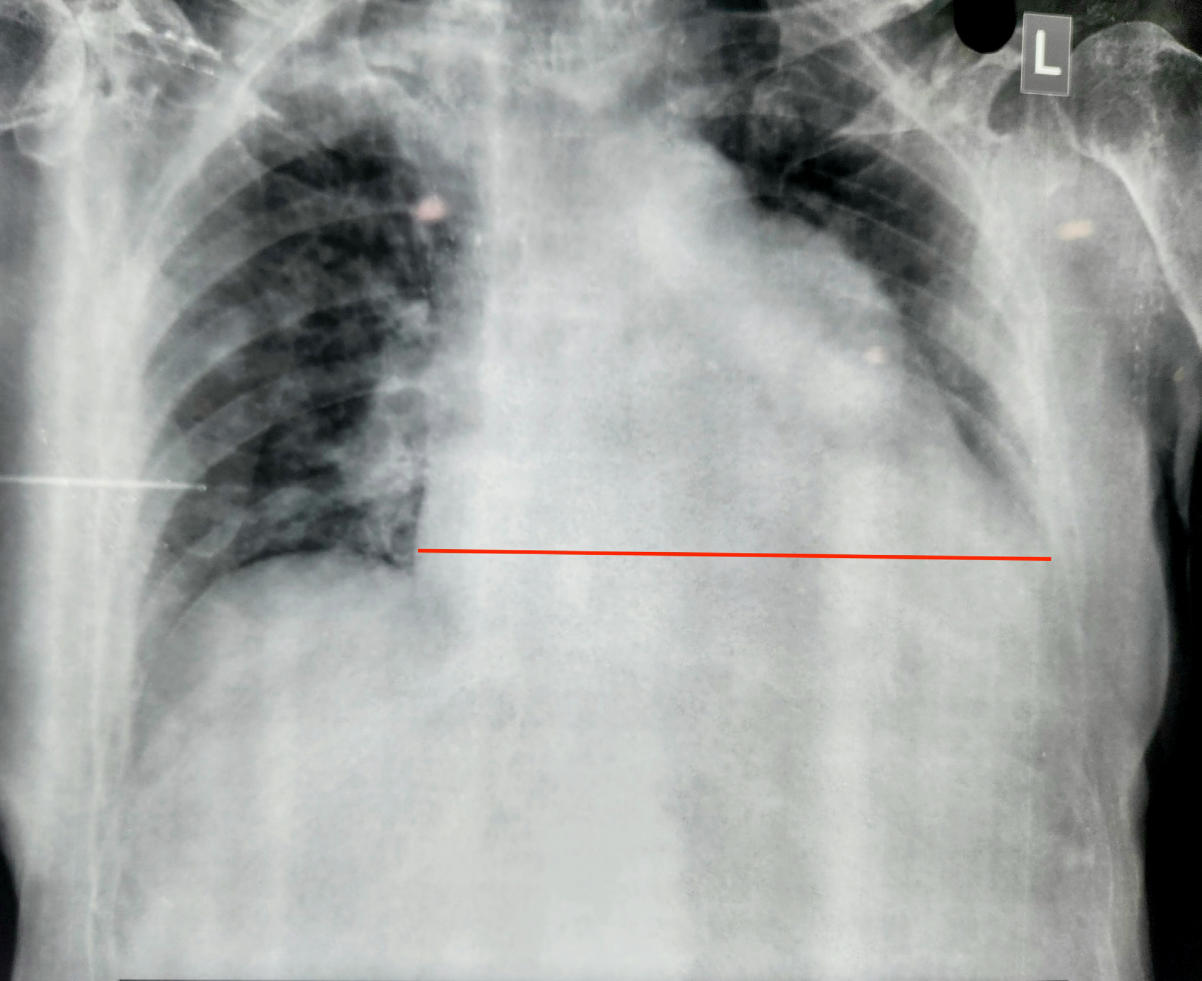
Chest X‐ray shows Cardiomegaly with a Cardiothoracic ratio >0.5 (marked by red line), enlarged cardiac silhouette, and pulmonary venous congestion.

**FIGURE 3:**
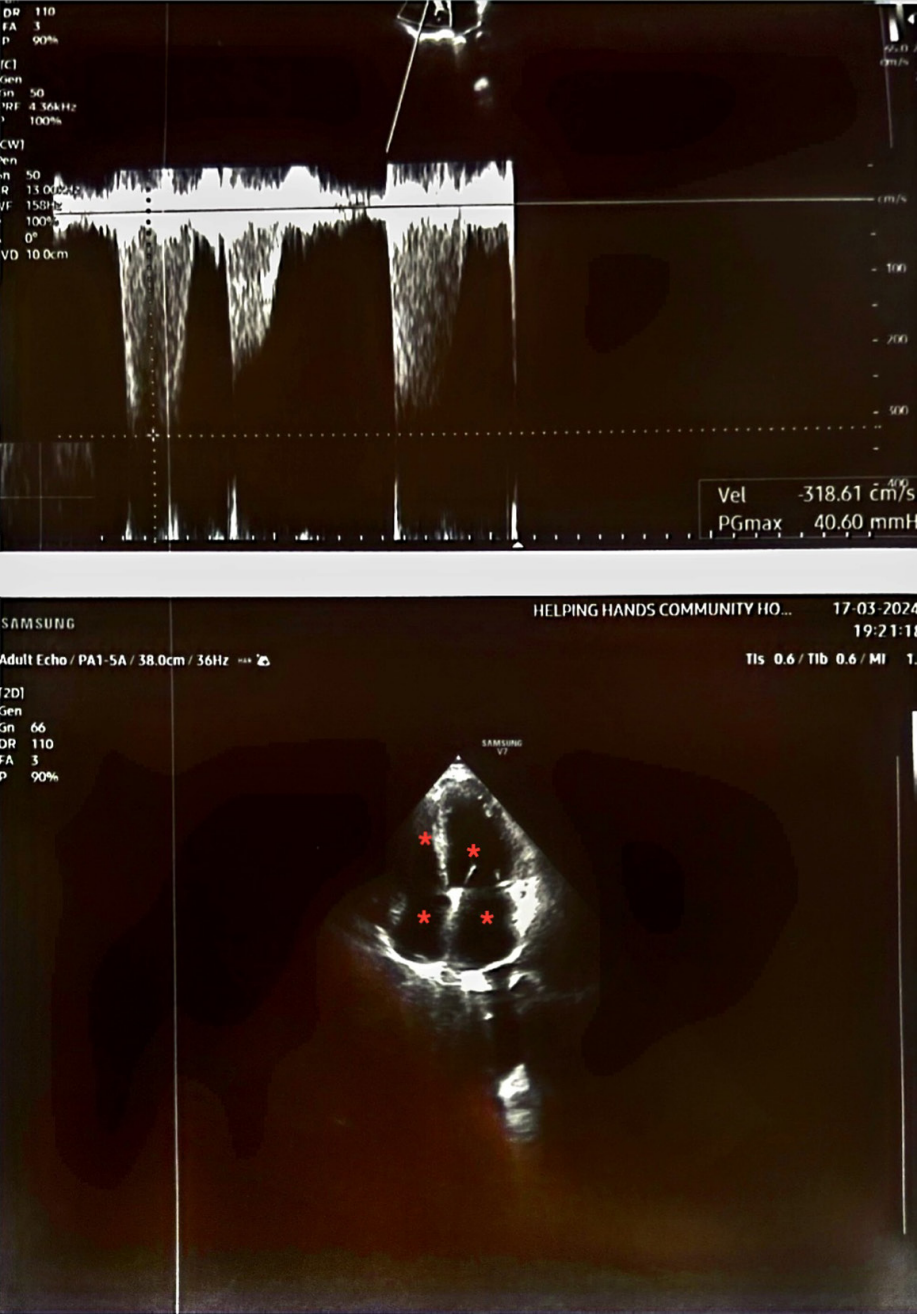
Echocardiography shows Dilated all chambers (marked by *), Global Left Ventricular wall hypokinesis, Moderate Mitral Regurgitation, Mild Aortic Regurgitation, Mild concentric Left Ventricular Hypertrophy, Moderate Tricuspid Regurgitation, Right Ventricular Systolic Pressure = 50 mmHg, Left Ventricular Diastolic Dysfunction, Moderate Left Ventricular Systolic Dysfunction, LVEF (Left Ventricular Ejection Fraction) = 30%–35%.

Findings of ECG, Chest X‐ray, and Echocardiography brain reports are described in Figure 1, Figure 2, and Figure 3.

On analysis of signs and symptoms and initial investigations, the patient was started on standard heart failure management with oxygen and diuretics. Even on 2 DOA (day of admission) patient's clinical signs and symptoms did not improve. However, in the 2nd DOA, she started to exhibit muscle spasm, tetany, circumoral numbness, and typical hypo‐calcemic signs of neuromuscular hyperexcitability: Chvostek, Trousseau positive signs.

On further analysis hypoparathyroidism, hypocalcemia, and vitamin D deficiency were seen. Lab reports are Total Calcium: 3.5 mg/dL, Albumin: 3.2 g/dL, PTH: <1 pg./L, Phosphorous: 7 mg/dL, Magnesium: 2 mg/dL, 25‐OH vitamin D: 10 ng/mL, and TSH: 2.5 mIU/L.

All other investigations for refractory heart failure like hypothyroidism were done and found to be non‐significant. Therefore, the patient was diagnosed with heart failure due to hypocalcemia due to primary hypoparathyroidism with vitamin D deficiency and dilated cardiomyopathy.

900 mg of IV calcium at 6 hours, followed by oral treatment – lactic calcium 6000 mg/day and calcitriol 0.5 μg per day was started. Clinical signs and symptoms of hypocalcemia and shortness of breath subsided on the 5th DOA.

The patient is discharged on the 10th DOA with 2000 mg/day of oral calcium and 1600 IU/daily of oral vitamin D. The association between correction of hypocalcemia and treatment of heart failure confirms the hypocalcemia as an etiology of heart failure.

### 1.3 Outcome and follow‐up

The patient is followed up monthly with echocardiograms and serum calcium measurements. On day 30 post‐admission (DOA), the patient did not report any issues. The echocardiogram showed an improved ejection fraction of 45 percent, with dilated ventricles and normal calcium levels. Repeat echocardiogram on the last follow‐up at 60 days post‐admission ejection fraction is 60 percent with normal left ventricular structure. The patient recovered completely with complete resolution of cardiac function on success replenishing calcium and vitamin D. Now, the patient is advised to follow up every 6 months and continue taking oral calcium and vitamin D.

## 2 DISCUSSION

Non‐ischemic heart muscle disorder with structural and functional abnormality characterized by left or biventricular dilatation and systolic dysfunction in the absence of other disorders is defined as DCM.^7^ The differential diagnosis of reversible cardiomyopathy includes hypothyroidism, hyperthyroidism, diabetes, pheochromocytoma, acromegaly, and beriberi. Hypoparathyroidism with hypocalcemia is another rare important condition that can result in reversible dilated cardiomyopathy.^8^ Although the prognosis is good many patients still die of advanced heart failure.^6^ Therefore, this case signifies the importance of considering hypo‐calcemic cardiomyopathy as a differential diagnosis of refractory heart failure which will help in early diagnosis and treatment and prevent conversion to advanced heart failure.

Hypo‐calcemic heart failure is common in infants due to severe vitamin D deficiency. In adults, although rare, Primary hypoparathyroidism is one of the most common causes of hypo‐calcemic cardiomyopathy.^9^ The other causes include hemosiderosis, thyroidectomy, parathyroidectomy, DiGeorge syndrome, and hemosiderosis.^3^ The most common manifestations of hypoparathyroidism and hypocalcemia are cerebral calcifications, cognitive deficit, cataracts, and epilepsy. Such a neurological manifestation was absent in our case; however, other signs of acute hypocalcemia, such as muscle spasms, perioral paresthesia, tetany, Chvostek sign, and Trousseau sign, were present. Hypo‐calcemic heart failure as the initial manifestation of primary hypoparathyroidism is a rare and unusual presentation and contributes to the limited literature on the subject.^16^

Hypocalcemia hampers cardiac contractility leading to reversible systolic dysfunction which could worsen upon the administration of drugs that block calcium such as calcium channel blockers.^2^ Also, Parathormone (PTH) appears to have a direct impact as it influences the l‐calcium ion channels within the heart muscle and also increases heart rate in newborn cells.^10^ In our case, we could not determine for how long primary hypoparathyroidism and hypocalcemia were present to have an effect on the heart. yet long‐term exposure to hypocalcemia is required to have an effect on structural damage in the myocardium as seen in other cases described in the literature review.^3^

In addition, calcium is also responsible for repolarization of cardiac myocytes which deficiency could cause QT prolongation and t‐wave inversion, as in our case where long QT was seen. Other cardiac manifestations include ventricular arrhythmias, refractory life‐threatening hypotension, and dilated cardiomyopathy.^11^ Moreover, hypocalcemia associated with cardiomyopathy could also be worsened by prolonged hypomagnesemia and low vitamin D levels.^2^

A higher risk of developing heart failure increased mortality and sudden death has been shown in human studies of vitamin D deficiency.^4^ In our case as well vitamin D deficiency was present which could have further complicated hypocalcemia‐associated cardiomyopathy and could have accelerated the process of development of hypo‐calcemic cardiomyopathy.

The heart failure in hypocalcemia is refractory to standard heart failure management, but rapidly responds to restoration of calcium levels in the blood like in our case. The prognosis of hypo‐calcemic cardiomyopathy is excellent with normalization of the left ventricular ejection fraction and non‐adherence could lead to recurrence of the conditions.

Very few cases reported complications of myocardial fibrosis and degeneration even after correction of calcium level.^6^ The ideal treatment of hypo‐calcemic cardiomyopathy is intravenous calcium followed by oral calcium.^12^, ^13^ Hypovitaminosis D, hypomagnesemia is commonly associated with hypo‐calcemic cardiomyopathy and its level should be tested, monitored, and corrected to avoid further complications.^14^ Calcium level will reach normal within a few days but it requires at least 6 months for restoration of cardiac function in our case only 3 months was required for restoration of cardiac function.^15^ The limited amount of available data and the absence of long‐term follow‐up mean there is insufficient information on long‐term complications and management protocols. Therefore, further studies are necessary.

## 3 IN CONCLUSION

Hypo‐calcemic cardiomyopathy is a rare cause of reversible dilated cardiomyopathy. Early diagnosis and treatment are necessary as it is reversible and can reduce morbidity and mortality. Therefore, hypocalcemia cardiomyopathy should be one of the differential diagnoses in case of refractory heart failure. In addition, hypomagnesemia, and vitamin D deficiency should always be monitored and treated, otherwise they could complicate the conditions.

## AUTHOR CONTRIBUTIONS

**Manish Kharel:** Conceptualization; resources; writing – original draft. **Anukul Subedi:** Resources; writing – original draft; writing – review and editing. **Md Fahad Hossain:** Software; supervision; writing – original draft; writing – review and editing.

## CONFLICT OF INTEREST STATEMENT

I declare that there is no conflict of interest regarding the publication of this paper. I, corresponding author on behalf of all contributing authors, hereby declare that the information given in this disclosure is true and complete to the best of my knowledge and belief.

## CONSENT

“I give written consent to use my clinical information for publication understanding that, it will be used only in educational publications intended for health professionals.”

## Data Availability

The data that support the findings of this study are available on request from the corresponding author. The data are not publicly available due to privacy or ethical restrictions.
